# From Iliac Fossa to Diaphragm: The Complex Presentation of a Ruptured Haemorrhagic Ovarian Cyst

**DOI:** 10.7759/cureus.70264

**Published:** 2024-09-26

**Authors:** Moustafa Elramlawy, Hany Abdelmasih, Karim Botros, Karim Hussien

**Affiliations:** 1 Emergency Department, Stoke Mandeville Hospital, Aylesbury, GBR; 2 Acute Medicine, Stoke Mandeville Hospital, Aylesbury, GBR; 3 Obstetrics Gynaecology, Poole General Hospital , University Hospitals Dorset NHS Foundation Trust, Bournemouth, GBR; 4 Trauma and Orthopaedics, Buckinghamshire Healthcare NHS Trust - Stoke Mandeville Hospital, Aylesbury, GBR

**Keywords:** abdominal pain in females, accident and emergency, gyne, haemorrhagic cyst of ovary, hemorrhagic cyst, hemorrhagic ovarian cyst, polycystic ovary syndrome (pcos)

## Abstract

A haemorrhagic ovarian cyst is a functional cyst resulting from bleeding into a follicular or corpus luteum cyst. It is a common gynaecological condition that often presents with acute pelvic pain, typically localized to one side, and may be accompanied by menstrual irregularities. Diagnosis is usually made via pelvic ultrasound, which typically reveals a complex cyst with characteristic features such as a reticular or 'lace-like' pattern. While most haemorrhagic cysts resolve spontaneously and require only conservative management, complications such as rupture or significant hemorrhage may necessitate surgical intervention. Occasionally, these cysts can present atypically, making diagnosis and management more challenging.

## Introduction

A haemorrhagic ovarian cyst is often asymptomatic and self-limiting. However, these cysts can rupture, releasing blood and fluid into the abdomen and pelvis, leading to potentially severe complications. Rupture can result in haemoperitoneum, compromised blood flow to vital organs and, in severe cases, sepsis. Although the symptoms of a haemorrhagic cyst often resemble those of other ovarian cysts, rupture can cause intense abdominal pain, irritation, nausea and vomiting. Immediate medical attention is required if a woman experiences persistent pain and ongoing bleeding, as these symptoms may indicate a need for urgent intervention [1].

## Case presentation

A 29-year-old multiparous female of Romanian descent presented to the emergency department (ED) with acute right iliac fossa pain that began a few hours earlier. The pain was sharp and progressively worsening. Upon arrival at the ED, the pain moved to the right hypochondrium, and the patient collapsed and briefly lost consciousness (LOC). She regained consciousness shortly after but remained in significant distress, reporting severe, persistent pain in the right hypochondrial area. No fever or other systemic symptoms were noted apart from dysuria, which was reported earlier that day. The patient reported no recent history of trauma or sexual activity in the last six months. Her menstrual history revealed irregular cycles. There was no other significant past medical or surgical history.

On assessment, her vital signs were as follows: blood pressure was 120/70 mmHg while lying down and 85/60 mmHg when standing; heart rate was 95 beats per minute, respiratory rate was 25 breaths per minute, and oxygen saturation was 99% on room air. Pupils were equal and reactive, with no focal neurological deficits observed. The abdomen was soft and non-tender all over; however, Murphy's sign was positive. No rigidity or guarding was noted.

Investigation

Full blood count, liver profile, kidney profile, and C-reactive protein levels all came back within normal ranges apart from mild elevation of white blood count and neutrophils (Table 1). A urine dipstick test was clear. Her blood beta-human chorionic gonadotropin (β-hCG) level was less than one. An abdominal ultrasound scan did not show any bleeding or collections.

**Table 1:** Laboratory results WCC: white blood cell count; β-hCG: beta-human chorionic gonadotropin

Test	Result	Reference	Unit of measurement
Haemoglobin	125	115 – 165	g/L
WCC	12.9	3.7 – 11	10^9^/L
Abs Neutrophils	10.0	1.7 - 7.5	10^9^/L
Platelets	194	150 – 450	10^9^/L
CRP	4.1	0 – 5	mg/L
Sodium	135	136 – 145	mmol/L
Potassium	3.5	3.5 - 5.1	mmol/L
Urea	4.5	2.5 - 6.7	mmol/L
Serum Creatinine	49	50 – 98	µmol/L
Alanine Transferase	17	10 - 35	U/L
Alkaline Phosphatase	59	40 – 150	U/L
Total Bilirubin	7	0 – 21	µmol/L
β-hCG	<1	<5 negative	IU/L
Amylase	68	25 – 125	U/L

Impression

Given the blood test results, normal urine dipstick, negative pregnancy test, and normal lactate levels in the venous blood gas, biliary colic was suspected as the primary differential diagnosis. The syncope was likely attributed to the pain, causing a vasovagal response. The patient was to complete her intravenous fluids, with a plan for discharge if she became pain-free. However, she unexpectedly developed a sudden onset of severe right-sided chest pain accompanied by shortness of breath. Despite her complaints, all her vital signs remained unchanged. An electrocardiogram, troponin levels, and chest X-ray showed no abnormalities. However, the patient continued to experience significant right-sided chest pain, and her Wells score for pulmonary embolism (PE) was calculated to be 3. Consequently, a D-dimer test was performed, yielding an elevated result of 3,450 µg/L (Table 2). Given the concerning symptoms and test results, a computed tomography pulmonary angiography (CTPA), along with a computed tomography (CT) scan of the abdomen and pelvis, was requested. Additionally, a full blood count (FBC) was reordered for further evaluation.

**Table 2:** Addendum blood test

Test	result	Reference	Unit of measurement
D-Dimers	3450 µg/L	0 – 500	µg/L
Troponin I	<2.0 ng/L	0 - 15.6	ng/L

Outcome

**Figure 1:**
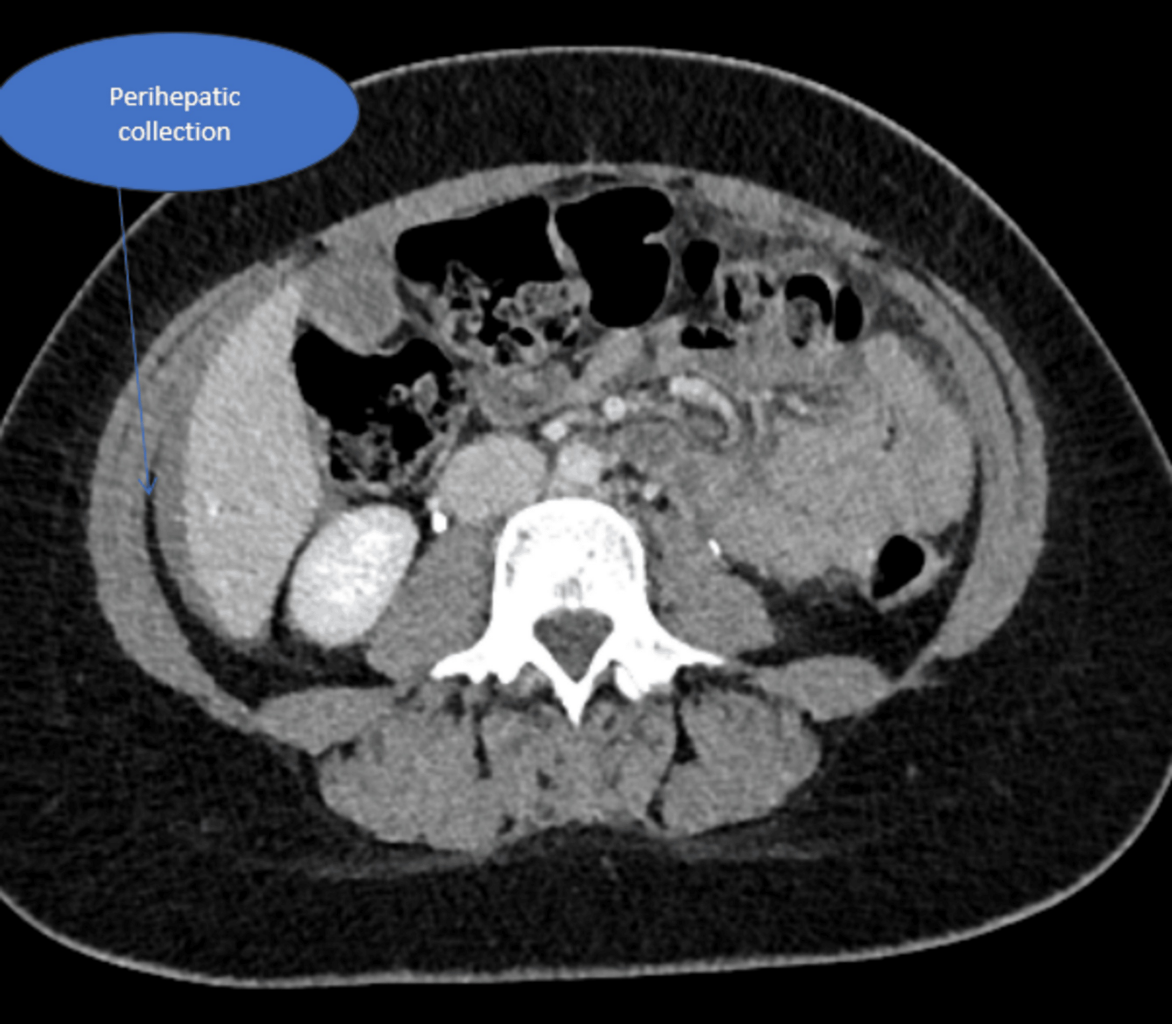
CT of the abdomen and pelvis shows bleeding around the liver

**Figure 2:**
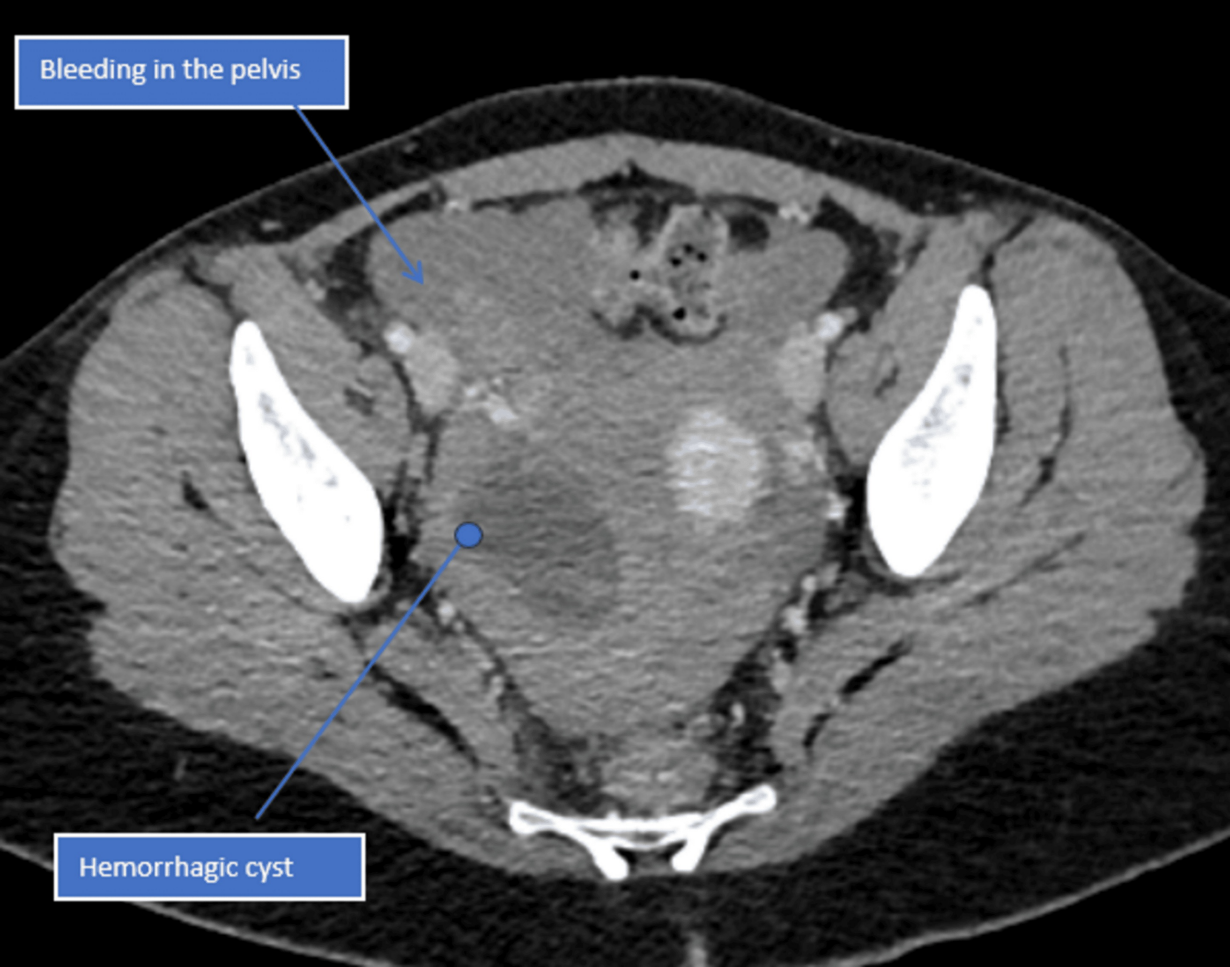
CT pelvis shows the cyst and the intra-abdominal bleed (arrows)

**Figure 3:**
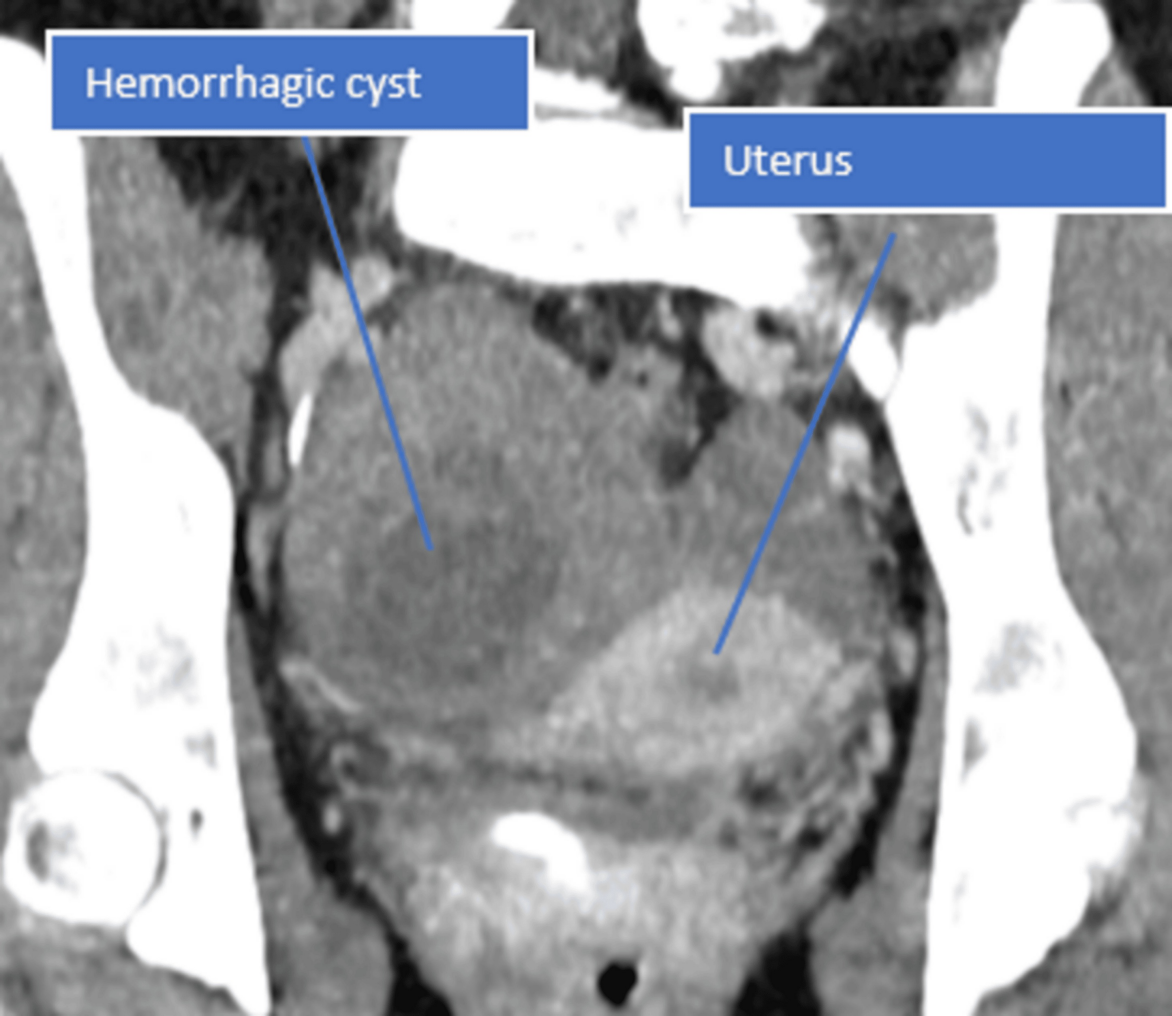
Longitudinal CT scan of the abdomen shows the haemorrhagic cyst

The CT report revealed hemoperitoneum (Figures 1-3), with no evidence of PE in the chest. The full blood count showed a significant drop in haemoglobin from 125 g/L to 56 g/L, indicating substantial internal bleeding. The patient was promptly taken to the anaesthetic room for preparation ahead of the operation. Aside from tachycardia secondary to hypovolemia, which was managed with a blood transfusion, she remained haemodynamically stable. A laparoscopic procedure was recommended due to its minimally invasive nature and faster recovery time. It was initially performed as a diagnostic procedure, with the intention to proceed therapeutically if necessary, and to minimize the risk of adhesions. The procedure successfully removed a large, ruptured haemorrhagic cyst via laparoscopy, with ovarian conservation. To manage the blood loss, two units of packed red blood cells were transfused intraoperatively. The patient showed excellent recovery through an enhanced recovery protocol, was discharged two days post-surgery, and returned home to care for her children.

## Discussion

The patient initially presented with right iliac fossa pain, which is a common location for issues related to the ovaries. The cyst eventually ruptured upon arrival at the hospital, leading to a collapse due to a sudden drop in blood pressure as a result of the internal bleeding. After being moved to a trolley, the patient's right iliac fossa pain subsided, as the ruptured cyst allowed blood to shift to the right hypochondrium. This shift in the location of the pain led to the clinical impression of a positive Murphy's sign (which is normally a sign of gall bladder inflammation) but in this case, the pain was because of the bleeding. Subsequently, the patient experienced a sudden onset of right-sided chest pain. This symptom was likely due to blood reaching the diaphragm and causing irritation, which initially prompted the clinical team to consider a pulmonary embolism. To accurately diagnose the patient's condition, both computed tomography pulmonary angiography and computed tomography of the abdomen and pelvis were necessary. The initial ultrasound scan did not help rule out other possibilities.

In the literature review, a case reported by Mantecon et al. described a 22-year-old woman with lower left abdominal pain, where imaging identified a ruptured hemorrhagic corpus luteum cyst, leading to internal bleeding. The condition was managed through successful laparoscopic removal of the cyst wall and accumulated blood [2]. In another report, clinical studies over 10 years show that symptoms of ruptured follicular and corpus luteum cysts can range from being unnoticed to causing haemorrhagic shock. The condition is more frequent in women over 30, with a 1:2 ratio of follicular to luteum cysts, primarily affecting the right ovary. Twenty per cent of patients were treated conservatively, and the preoperative and intraoperative diagnoses matched in 92% of cases [3]. In another report, CT imaging is highlighted as valuable in diagnosing bleeding from ovarian cyst rupture. It can show varying effusion densities in the pelvic cavity and upper abdomen, reveal the cystic mass even amid a large effusion and identify irregular cystic walls indicating rupture, as well as direct extravasation and collection of contrast material in the pelvic cavity. This information is crucial when ultrasound results are negative or unclear. Overall, CT imaging provides essential diagnostic insights in such cases [4]. A ruptured ovarian cyst can cause massive hemoperitoneum, mimicking conditions like ectopic pregnancy. Radiologists need to consider this possibility in women of childbearing age with pelvic pain, as misdiagnosis is common. Large amounts of complex intraperitoneal fluid are key indicators [5]. Ovarian haemorrhage from the corpus luteum, whether associated with menstruation or pregnancy, can be a life-threatening surgical condition occurring at all stages of a woman's reproductive life. Most ruptures occur in the right ovary and can be misdiagnosed as appendicitis [6,7].

Learning points

Firstly, this case emphasized the importance of considering gynaecological emergencies in women who present with acute abdominal pain, even when initial symptoms or investigations suggest other diagnoses. Haemorrhagic ovarian cysts, in particular, can mimic a range of conditions, making it crucial to maintain a broad differential diagnosis, especially in women of reproductive age. Secondly, this case demonstrated how the shifting nature of pain and symptoms, including syncope and right-sided chest pain, can complicate the diagnostic process. The ruptured haemorrhagic cyst in this case presented with symptoms that closely resembled biliary colic and pulmonary embolism, leading to initial diagnostic confusion. Thirdly, this case underscored the importance of vigilant monitoring and reassessment in clinical practice. Normal initial investigations, such as blood tests and ultrasound scans, do not necessarily rule out serious pathology. Continuous reassessment is essential, particularly when symptoms evolve or persist. Additionally, the role of advanced imaging in diagnosis is highlighted, as the computed tomography scans were crucial in identifying the hemoperitoneum and the underlying cause, which was missed on the initial ultrasound. This case reinforced the value of using appropriate imaging modalities when clinical suspicion remains high. Lastly, the case emphasizes that early surgical intervention can be life-saving. Timely recognition and management of a ruptured haemorrhagic cyst are vital to prevent life-threatening complications such as severe haemorrhage. The need for quick decision-making and intervention in acute settings is clearly demonstrated here.

## Conclusions

This case highlighted the complexity involved in diagnosing haemorrhagic ovarian cysts, particularly when they present with atypical symptoms that mimic other conditions such as biliary colic or pulmonary embolism. The initial presentation of right iliac fossa pain, followed by syncope and shifting pain patterns, underscored the dynamic nature of a ruptured haemorrhagic cyst and the challenges it poses in clinical diagnosis. The delayed recognition of haemoperitoneum, ultimately confirmed by imaging and a significant drop in haemoglobin, emphasizes the importance of maintaining a high index of suspicion for gynaecological emergencies in women of reproductive age, even when initial investigations appear unremarkable. In this case, prompt surgical intervention proved to be life-saving, highlighting the need for timely and thorough evaluation in similar clinical scenarios. This case also illustrated the necessity for clinicians to consider a broad differential diagnosis when assessing acute abdominal pain, particularly in women, to ensure appropriate and effective management.

In conclusion, this case illustrated the need for a comprehensive understanding of the physiological changes that occur following a cyst rupture. The movement of blood within the abdomen post-rupture can lead to pain in different locations, creating diagnostic challenges. Clinicians must be aware of how intraperitoneal bleeding can present with varying symptoms, depending on patient positioning and the effects of gravity.
